# Gender norms and modern contraceptive use in urban Nigeria: a multilevel longitudinal study

**DOI:** 10.1186/s12905-018-0664-3

**Published:** 2018-10-29

**Authors:** Chinelo C. Okigbo, Ilene S. Speizer, Marisa E. Domino, Sian L. Curtis, Carolyn T. Halpern, Jean C. Fotso

**Affiliations:** 1 Department of Maternal and Child Health, Gillings School of Global Public Health, University of North Carolina at Chapel Hill, Chapel Hill, North Carolina USA; 2 Measurement, Learning & Evaluation Project, Carolina Population Center, University of North Carolina at Chapel Hill, Chapel Hill, North Carolina USA; 3 Department of Health Policy and Management, Gillings School of Global Public Health, University of North Carolina at Chapel Hill, Chapel Hill, North Carolina USA; 4 Cecil G. Sheps Center for Health Services Research, University of North Carolina at Chapel Hill, Chapel Hill, North Carolina USA; 5 Innovations for Maternal, Newborn & Child Health, Concern Worldwide USA, New York, USA

**Keywords:** Gender norms, Modern contraception, Longitudinal data, Multilevel models, Urban Nigeria

## Abstract

**Background:**

Evidence suggests that gender equality positively influences family planning. However, the evidence from urban Africa is sparse. This study aimed to examine the association between changes in gender norms and modern contraceptive use over time among women in urban Nigeria.

**Methods:**

Data were collected in 2010/2011 from 16,118 women aged 15–49 living in six cities in Nigeria (Abuja, Benin, Ibadan, Ilorin, Kaduna, and Zaria) and again in 2014 from 10,672 of the same women (34% attrition rate). The analytical sample included 9933 women living in 480 neighborhoods. A four-category outcome variable measured their change in modern contraceptive use within the study period. The exposure variables measured the changes in the level of gender-equitable attitudes towards: a) wife beating; b) household decision-making; c) couples’ family planning decisions; and d) family planning self-efficacy. Multilevel multinomial logistic regression models estimated the associations between the exposure variables at the individual and neighborhood levels and modern contraceptive use controlling for the women’s age, education, marital status, religion, parity, household wealth, and city of residence.

**Results:**

The proportion of women who reported current use of modern contraceptive methods increased from 21 to 32% during the four-year study period. At both surveys, 58% of the women did not report using modern contraceptives while 11% reported using modern contraceptives; 21% did not use in 2010/2011 but started using by 2014 while 10% used in 2010/2011 but discontinued use by 2014. A positive change in the gender-equitable attitudes towards household decision-making, couples’ family planning decisions, and family planning self-efficacy at the individual and neighborhood levels were associated with increased relative probability of modern contraceptive use (adoption and continued use) and decreased relative probability of modern contraceptive discontinuation by 2014. No such associations were found between the individual and neighborhood attitudes towards wife beating and modern contraceptive use. Accounting for the individual and neighborhood gender-equitable attitudes and controlling for the women’s demographic characteristics accounted for 55–61% of the variation between neighborhoods in the change in modern contraceptive use during the study period.

**Conclusion:**

Interventions that promote gender equality have the potential to increase modern contraceptive use in Nigerian cities.

**Electronic supplementary material:**

The online version of this article (10.1186/s12905-018-0664-3) contains supplementary material, which is available to authorized users.

## Background

The fertility rate in Nigeria, the most populous country in Africa, remains high; a woman living in Nigeria is estimated to have 5.5 children during her lifetime [1]. A recent study estimated that about one quarter of all pregnancies that occur in Nigeria annually are mistimed or unwanted [2] and about one-half of unintended pregnancies end in abortion [2]. Unintended pregnancy contributes significantly to maternal deaths through increased prevalence of unsafe abortions and/or complications during pregnancy [2–4]. According to the World Health Organization, Nigeria was the highest contributor to global maternal mortality in 2015 [5]. An estimated 58,000 maternal deaths occurred in 2015 giving a maternal mortality ratio (MMR) of 814 deaths per 100,000 live births [5]. To put these figures in perspective, the current lifetime risk of dying from a pregnancy-related cause is 1 in 22 in Nigeria compared to 1 in 3800 and 1 in 5800 in the United States of America and the United Kingdom, respectively [5]. A 2012 study revealed that about one in five maternal deaths in Nigeria were averted by family planning – a primary prevention strategy for maternal mortality [4]. In addition to preventing pregnancy-related morbidity and mortality, family planning also improves women’s empowerment through increased education and subsequent engagement in the workforce [6–9].

Not all women who want to avoid a pregnancy practice family planning. According to the Nigeria Demographic and Health Surveys (NDHS), the contraceptive prevalence rate (CPR), that is the percentage of women aged 15–49 using contraception, has been on the rise since 1990. Among married women, the CPR increased from 6% in 1990 to 15% in 2013 [1, 10]. The majority of that increase was in the use of modern contraceptive methods^1^ (hereafter referred to as modern contraceptives), which increased from 3.5 to 9.8% from 1990 to 2013. Most of that increase happened in the 1990s – 3.5% in 1990 to 8.6% in 1999 [10]. Since the 2000s, the CPR in Nigeria has stalled at approximately 10% for modern contraceptives and 5% for traditional contraceptives (i.e. withdrawal and periodic abstinence). Thus, there seems to be barriers to the practice of family planning in Nigeria.

According to the 2008 NDHS, about 40% of married women who were not using a contraceptive method cited opposition to family planning as their reason for not practicing contraception [11]. This opposition to family planning was disaggregated into opposition from the women (21%), male partners (10%), and others including religious institutions (9%). Sedgh & Hussian (2014) found similar results among Nigerian women with an unmet need for family planning [12]. Stephenson and colleagues (2007) in their study of modern contraception in sub-Saharan Africa found that women were more likely to use modern contraceptives if they perceived that other community members approved of family planning [13]. Likewise, a recent study by Gueye and colleagues (2015) found that women who feared side effects and health risks of modern contraceptives or who lived in communities where such fears were prevalent were less likely to use modern contraceptives [14]. Women living in societies where women’s status is low often lack the power to make reproductive decisions including family planning use, seeking antenatal care, or delivering in a health facility [15–21].

### Gender equality and Women’s empowerment

The United Nations defines gender as the “socially constructed roles and relationships, attitudes, behaviors, values, relative power, and influence that society ascribes to the two sexes on a differential basis” [22]. Gender differs from sex (the biological and genetic characteristics of an individual) in that gender is acquired and varies over time, within, and across cultures. Attitudes towards gender roles and relationships, referred to as gender norms, operate at multiple levels of the socioecological system – individual, household, neighborhood, and community – leading to social conformity [17, 22]. Thus, in societies where the gender norms are inequitable towards women, women who do not conform to these norms often suffer adverse outcomes [23]. Inequitable gender norms may directly lead to negative health outcomes. For example, female circumcision, a sociocultural practice in Nigeria to curtail women’s sexuality, is associated with adverse obstetric outcomes such as severe bleeding during childbirth [24]. Also, inequitable gender norms indirectly influence health outcomes through women’s lack of decision-making power. Hence, improving gender-equitable norms may lead to women’s empowerment.

Women’s empowerment continues to be a focus for global health and development. At the turn of the millennium, 172 countries signed the United Nations’ Millennium Declaration to achieve eight Millennium Development Goals (MDGs) by 2015 among which included Goal 3 (promote gender equality and empower women) and Goal 5 (improve maternal health) [25]. By the 2015 deadline, the United Nations reported that many countries made progress towards achieving these goals; however, gender inequality and maternal mortality persisted especially among the poorest and most vulnerable populations [26]. A new set of goals to be achieved by 2030 – Sustainable Development Goals (SDGs) – were set at the end of 2015 to maintain the progress made during the MDG-era and to work towards ending the gender, wealth, and health inequalities [26]. More research is needed to better understand the pathways through which gender equality influence women’s health. This study aimed to examine the relationships between changes in gender-equitable attitudes and changes in modern contraceptive use over time among reproductive-aged women living in select Nigerian cities. Four dimensions of gender-equitable attitudes were assessed: attitudes towards wife beating, household decision-making, couples’ family planning decisions; and family planning self-efficacy.

### Theoretical framework linking gender-equitable norms and modern contraceptive use

This study is informed by the Theory of Gender and Power developed by Robert Connell in 1987 [27] and adapted by Wingood & DiClemente in 2000 [28]. The theory posits that three social structures (division of labor, division of power, and structure of cathexis) exist at the societal level and together shape the gendered relationships between men and women at the interpersonal and individual levels [27–29]. These three interrelated structures are preserved in society through sociocultural mechanisms that continuously segregate power and assign roles and responsibilities to the different genders. According to the Wingood & DiClemente adaptation of the theory, gender-based inequalities created by these three social structures at the societal and community levels lead to increased risk factors and behaviors among the lower status gender at the interpersonal and individual levels [28, 29].

The division of labor describes the allocation of unequal educational and occupational opportunities to women in relation to men, limiting women’s economic potential [28–30]. For example, women are constrained to institutions that cater to nurturing such as childcare and domestic work. These types of work are often uncompensated or undercompensated leading to economic inequalities – leaving these women to rely on men for financial sustenance. As the economic inequality between men and women widens, women experience more economic risk factors (e.g. inability to pay for health services), which then lead to adverse health outcomes. The division of power refers to the allocation of more power to one gender over the other. Power is defined in this context as the capability to influence one’s actions and/or the actions of others [28]. In patriarchal societies, men exercise more power over their female partners, who may need to seek permission from the men to engage in activities such as visiting family/friends. As the power inequality favoring men increases, women tend to experience more risk factors that predispose them to adverse outcomes [17]. For instance, a pregnant woman in labor waiting for the husband’s permission to go to the hospital may delay receiving a medical intervention without which her health or that of her baby may be jeopardized. The structure of cathexis in the Theory of Gender and Power describes what the society deems as appropriate behaviors for men and women [28, 29]. In reproductive health, this structure describes the cultural and religious norms that impose strict sexual responsibilities on women (e.g. women should have sex to satisfy the desires of their male partners and for procreation, not for pleasure). This sexual inequality results in risky behaviors that negatively affect women’s health, e.g. coerced sex that may lead to unintended pregnancies. All three structures are interrelated and together result in risk factors that increase the prevalence of adverse outcomes. For example, women who live in communities with religious and/or cultural restrictions on female sexuality (structure of cathexis) may be more likely to depend on their male partners for finances (division of labor) and may need permission from them to seek health services (division of power). Such women may experience increased economic, psychologic, and behavioral risk factors that endanger their health.

**Fig. 1:**
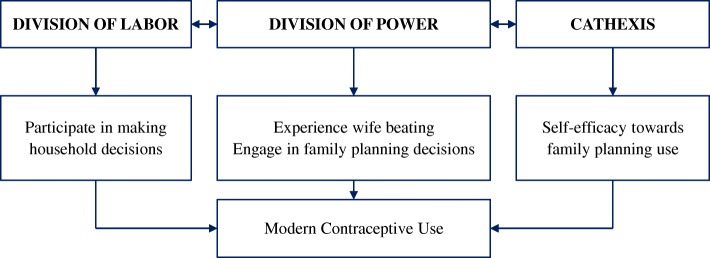
The Theory of Gender and Power adapted to the study of modern contraception

The Theory of Gender and Power was applied in this study to describe the relationships between the social structures and modern contraceptive use in urban Nigeria (See Fig. 1). Under the division of labor, it is hypothesized that women who experience economic inequality will be unable to afford family planning services if they desired to practice contraception. In this study, the attitudes towards women’s participation in household decision-making were included to reflect inequalities under the division of labor. Researchers have documented that women who participate in household decision-making have higher odds of using modern contraceptives compared to their less empowered counterparts [16, 18, 31, 32]. Under the division of power, it is hypothesized that women who lack authority over their life choices or those who experienced gender-based violence will be less empowered to make reproductive health decisions such as modern contraceptive use. The attitudes towards wife beating and attitudes towards couples’ family planning decisions were included in this study to reflect inequalities under the division of power. A recent study in southern Nigeria found that about two-thirds of their respondents did not support women having independent rights to contraceptive method choice, adoption, and use [33]. Without such rights, women were less likely to practice family planning. Thus, under the structure of cathexis, attitudes towards self-efficacy to family planning use were hypothesized to positively influence women’s use of modern contraceptives.

A recent literature review of 60 studies from developing countries that examined the association between women’s empowerment and fertility reported that the majority of the studies were from South Asia, relied on cross-sectional data, focused on married women, with only a handful of the studies accounting for the influence of the community-level women’s empowerment on fertility [18]. Our study fills the current gap in the literature by providing information on multiple levels of influence of gender norms on the use of modern contraceptives from a longitudinal sample of both married and unmarried women living in urban areas of Nigeria. Given the multidimensionality of the factors under the three structures in the Theory of Gender and Power, multiple aspects of gender equality were assessed to provide a better contextual picture of the effects of gender norms on modern contraceptive use over time. Our study hypothesized that the women who hold gender-equitable attitudes, compared to those who do not, will have higher probability of adopting and/or continuing use of modern contraceptives over time. Also, women who live in neighborhoods where other women hold gender-equitable attitudes will have a higher probability of adopting and/or continuing use of modern contraceptives over time compared to those who live in neighborhoods where other women do not hold such gender-equitable attitudes.

## Methods

### Study design and sample

Our study used de-identified longitudinal data collected in Nigeria by the Measurement, Learning & Evaluation (MLE) project for the purposes of evaluating a family planning program – the Nigeria Urban Reproductive Health Initiative (NURHI). NURHI was part of a five-year multi-country multi-strategy family planning program, the Urban Reproductive Health Initiative, funded by the Bill & Melinda Gates Foundation and implemented in select urban areas of four countries – Nigeria, Kenya, Senegal, and India [34]. NURHI aimed to increase the use of modern contraceptives in six purposively selected cities: Abuja, Benin, Ibadan, Ilorin, Kaduna, and Zaria. The key strategies implemented by NURHI included: strengthening family planning service delivery in both public and private health facilities, generating demand for family planning services through community engagement and multimedia campaigns, building a supportive environment for family planning through advocacy, and using monitoring and evaluation data to improve program activities. Information about NURHI can be found on their website [http://www.nurhitoolkit.org]. To evaluate NURHI, the MLE project conducted longitudinal data collection at two-year intervals from 2010 to 2014. Our study used data collected from a representative sample of reproductive-aged women interviewed during the first survey in 2010/2011 (baseline) and who were followed over the four-year period and re-interviewed during the final survey in 2014 (endline). The women at baseline were selected using a two-stage cluster sampling design. A sampling frame based on the most recent census in Nigeria (2006 census) was used to select a sample of enumeration areas. Enumeration areas are subdivisions of localities, which are the smallest administrative units. These enumeration areas were used as the primary sampling units and are hereafter termed clusters. In the first stage of sampling, a random sample of clusters was selected in each city. The number of clusters selected ranged from 74 in Zaria to 102 in Ibadan with a total of 491 clusters in the six cities. At the second stage of sampling, a random sample of 41 households was selected in each cluster. All women aged 15–49 years who resided in the selected households or were visitors present on the night before the survey were eligible to be interviewed.

The baseline survey was conducted between October 2010 and April 2011. A total of 16,144 women from about 16,000 households located in 491 clusters completed the survey at baseline giving a 95% response rate. Detailed information about the baseline survey is published elsewhere [35]. Four years after the baseline survey, the women who were not visitors to the households at the time of baseline survey were tracked and, if found, were re-interviewed (endline survey). The endline survey was conducted in all cities between June and October 2014. Of the 16,118 women who were not visitors to the households at baseline, 10,672 women in households located in 489 clusters were found and completed the endline survey – giving a 66% response rate. About one-third of the women were lost to follow-up because they died, moved out of the study cities, or could not be found. Detailed information about the endline survey is published elsewhere [36]. An attrition analysis showed that women lost to follow up were younger, single, and in the poorest wealth group [37].

**Fig. 2:**
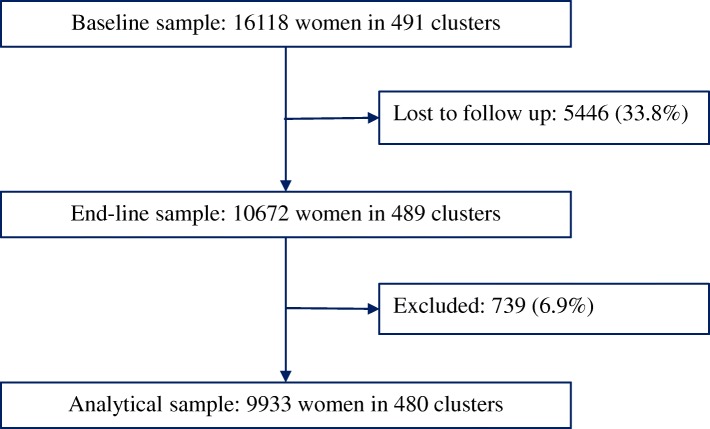
Sample selection flowchart

The analytical sample for this study was restricted to women aged 15–49 interviewed at both surveys. Women were excluded if they were aged more than 49 years at endline (*n* = 592) or had missing data on any of the included variables (*n* = 120). Clusters with fewer than five women were dropped (9 clusters with 27 women) as multilevel statistical models are known to provide reliable and valid estimates if the groups have at least five observations [38]. Thus, the analytical sample consisted of 9933 women living in 480 clusters (Fig. 2).

### Measures

#### Outcome variable

The outcome variable was a four-category variable indicating the change, or lack thereof, in use of modern contraceptives between the two surveys. The modern contraceptive use variable was created from two sequential questions asked at both surveys: a) “are you (or your partner) currently doing something or using any method to delay or avoid getting pregnant?” and b) “which method(s) are you (or your partner) currently using?” Women were coded as having a value of ‘1’ if they responded ‘yes’ to the first question and, in response to the second question, chose any of these contraceptive methods identified by the World Health Organization as modern contraceptives: daily pill, injection, implant, intrauterine device, sterilization (female or male), diaphragm, emergency pill, spermicide (gels or foams), condom (male or female), and lactational amenorrhea [39]. Women who did not answer affirmatively to the first question or did not choose any of the aforementioned methods for the second question were coded as having a value of ‘0’. Thus, women were recoded as ‘users’ or ‘non-users’ of modern contraceptives at each of the surveys. A categorical outcome variable with four response options was then created to reflect the change, or lack thereof, in use of modern contraceptives within the study period. The response options included the following groups of women: ‘non-users’, ‘users’, ‘adopters’, and ‘discontinuers’. The non-users were women who reported not using a modern contraceptive method at both surveys while the users were women who reported using a modern contraceptive method at both surveys. The adopters were women who reported not using a modern contraceptive method at baseline but reported using a modern contraceptive method at endline while the discontinuers were women who reported using a modern contraceptive method at baseline but reported not using a modern contraceptive method at endline.

#### Exposure variables

To evaluate the association between gender-equitable attitudes and modern contraceptive use, four exposure variables were utilized to represent attitudes toward: wife beating, household decision-making, couples’ family planning decisions, and family planning self-efficacy. Evidence suggests that attitudes in these four areas represent different aspects of gender-equitable attitudes and may differentially affect modern contraceptive use.

#### Attitudes towards wife beating

During each survey, the women were asked whether they believed a husband is justified to beat his wife using seven scenarios: going out without telling him, neglecting the house or children, arguing with him, refusing to have sex with him, cooking food improperly, suspecting she is unfaithful, and refusing to have another child. The response options were ‘yes’, ‘no’, and ‘don’t know’. The Cronbach’s alpha was 0.94 at baseline and 0.80 at endline. The responses were first dichotomized to ‘1’ if the woman answered ‘no’ or ‘0’ if she answered ‘yes’ or ‘don’t know’. The dichotomized responses were summed to scores ranging from 0 to 7. Increasing score reflects more gender-equitable attitudes towards wife beating.

#### Attitudes towards household decision-making

The women were also asked their opinions on who they thought should have a greater say in four scenarios: making small household purchases, making large household purchases, deciding when to visit family/friends, and when/where to seek medical care for their own health. The response options were ‘husband’, ‘wife’, ‘both’, and ‘don’t know’. The Cronbach’s alpha was 0.84 at baseline and 0.68 at endline. The responses were first dichotomized to ‘1’ if wife/both or ‘0’ if husband/don’t know. The dichotomized responses were summed to scores ranging from 0 to 4; increasing score reflects gender-equitable attitudes towards household decision-making.

#### Attitudes towards couples’ family planning decisions

This variable was created from responses to a set of nine statements to which the women were asked to state whether they agreed or disagreed. The statements included: a) the husband should be the one to decide whether the couple should use a family planning/birth spacing/child spacing method; b) couples who practice family planning have a better quality of life than those who do not; c) husbands and wives should discuss family planning; d) men should not allow their wives to use family planning; e) a woman who uses family planning without her husband’s knowledge should be punished; f) a woman who has no children is not complete/fulfilled; g) a man who has no children is not complete/fulfilled; h) a woman should continue bearing children until she has at least one son; and i) a woman should continue bearing children until she has at least one daughter. The Cronbach’s alpha was 0.89 at baseline and 0.66 at endline. The responses to statements a, d-i were recoded to ‘1’ if strongly disagree/disagree and ‘0’ otherwise while the responses to statements b and c were recoded to ‘1’ if strongly agree/agree and ‘0’ otherwise. The dichotomized responses were summed to scores ranging from 0 to 9. The higher the score, the more gender-equitable the attitudes towards couples’ family planning decisions are.

#### Attitudes towards family planning self-efficacy

The women were also asked to state whether they agreed or disagreed with certain statements that assessed their perception about their ability to practice family planning when they wanted. The statements were: you could start a conversation with your partner about family planning; you could convince your partner that you should use a method of family planning; you could get to a place where family planning methods are offered if you decided to use one; you could obtain a family planning method if you decided to use one; you could use a family planning method even if your partner doesn’t want you to; you could use a method of family planning if none of your friends or neighbors uses one; you could use a family planning method even if your religious leader did not think you should use one; and you could continue to use a family planning method if you experience some side effects. The Cronbach’s alpha was 0.93 at baseline and 0.86 at endline. The responses were first dichotomized to ‘1’ if strongly agree/agree or ‘0’ if strongly disagree/disagree. The dichotomized responses were then summed to scores ranging from 0 to 8 with increasing score reflecting increasing family planning self-efficacy.

The exposure variables were further categorized as ‘low level’ or ‘high level’ based on the median scores for each index at baseline. The baseline median scores for the attitudes towards wife beating, household decision-making, couples’ family planning decisions, and family planning efficacy were 7, 2, 6, and 5, respectively. Thus, the variables were dichotomized into ‘low levels’ if the score were lower or equal to the median scores and ‘high levels’ otherwise for all measures except for the attitudes towards wife beating score that was classified as ‘low level’ if the score was 0–6 or ‘high level’ if the score was equal to 7. These attitudes were also measured at the neighborhood (cluster) level using a three-step method: 1) the individual scores were aggregated to the cluster level; 2) the index woman’s score was subtracted from the cluster-level score; and then 3) the cluster-level score was divided by the number of women in that cluster minus one. The resultant neighborhood-level score represented the mean score for the attitudes of the other women in the same cluster. Just like the individual scores, the scores at the neighborhood-level were dichotomized into ‘low level’ or ‘high level’ based on the individual-level baseline median score. Finally, categorical variables measuring the change, or lack thereof, in the level of the attitudes between baseline and endline surveys were created for the individual and neighborhood levels. The categories included: ‘low to low’, ‘low to high’, ‘high to low’, and ‘high to high’ levels indicating the change from baseline to endline surveys.

### Control variables

The baseline socio-demographic factors were included as control variables. The women’s ages, which ranged from 15 to 49 years, were grouped into ‘15–24’, ‘25–34’, and ‘35–49’ age groups and their educational level were grouped into ‘none/Quranic’, ‘primary’, ‘secondary’, and ‘higher’ education. Religion was measured as Catholic, other Christian, Muslim, and no religion, which was recoded into ‘Muslim’ versus ‘non-Muslim’. The number of children the women had ever birthed was categorized into ‘none’, ‘1–4’, or ‘5 or more’ children. The women’s household wealth index was calculated using principal components analyses of several household items including, but not limited to, electricity, source of drinking water, toilet facility, land/livestock ownership, and type of household building materials. The weighted household index score was divided into quintiles (poorest, poor, middle, rich, and richest) and used as a proxy for household economic status. Marital/union status, which was measured as never married, currently married, living with a man, widowed, divorced, or separated was dichotomized into ‘currently married or cohabiting’ versus ‘not currently married or cohabiting’. Since a change in union status is likely to be associated with a change in fertility intention, the union status variable was recoded into a categorical variable that reflected the change in status between surveys. The categories were ‘never in-union’, ‘became in-union’, and ‘ever in-union’. The variable for the city of residence had six categories: Abuja, Benin, Ibadan, Ilorin, Kaduna, and Zaria. The choice of the functional forms of all the variables used in this study (continuous, categorical, or binary) were made based on the form that fit the data the best using model fit indices.

### Statistical analyses

All statistical analyses were conducted in Stata version 14 and were weighted to account for the study design and non-response [40]. Descriptive analyses were first conducted to provide information about the socio-demographic distribution of our study sample, together with the distribution of the exposure and outcome variables. Then, the multilevel multinomial logistic regression models were run to test the relationships between the individual and neighborhood gender-equitable attitudes and the modern contraceptive use pattern. The rationale for using multilevel models was based on the hierarchical data structure (women nested in neighborhood clusters) and we hypothesized that some effects will operate at the neighborhood level through peer influence. Multilevel models simultaneously run regression models for each data level taking into account the lack of independence of the nested observations and residuals. Additionally, multilevel models partition the variance in the outcome variable to that due to the individual versus cluster levels. For this study, the outcome variable was included at the individual level while the exposure variables were included at the individual and neighborhood levels. The control variables were also included at the individual level.

The multilevel modeling was conducted using a user-written Stata command ‘gllamm’ which stands for Generalized Linear Latent and Mixed Models. GLLAMM are a class of multilevel models that estimate fixed and random effects of various types of outcome variables including continuous, count, binary, ordered and unordered categorical variables [41–43]. In this study, two-level multinomial logistic regression models were run with discrete factor approximation, which give maximum likelihood semi-parametric estimators that are consistent and asymptotically efficient under the model’s assumptions [44]. An intercept-only (null) model was run to test the null hypothesis that there was no between-cluster variation in the modern contraceptive use pattern during the study period. The estimate from this model was used to calculate the proportion of the variance in the outcome variable that is attributable to the cluster level. This proportion is estimated by calculating the intra-class correlation coefficient (ICC), which in this study describes the extent to which women in the same neighborhood are similar to each other relative to women in different neighborhoods. Separate multivariate models containing the individual and neighborhood exposure variables together with the control variables were then run for each of the four dimensions of gender-equitable attitudes. Since the coefficients from multinomial logistic models cannot be directly interpreted, post-estimation exponentiation of the coefficients was performed to produce odds ratios, interpreted as relative probabilities. The variance inflation factor assessed multi-collinearity of variables and was found to be below the cut-off point of 10 in all models. Post-estimation goodness-of-fit tests were also conducted. Akaike Information Criteria and Bayesian Information Criteria values of the individual-only models and full models were compared to the null model. Likelihood Ratio tests comparing the full models to the null model and to the model containing only the individual-level exposure variables were also conducted.

### Ethical approval

The ethical approval for the study was obtained from the Nigeria Health and Research Ethics Committee and the University of North Carolina at Chapel Hill Institutional Review Board. Verbal informed consent was obtained from all respondents prior to each round of study participation. Women who were aged less than 18 years (ages 15–17) were considered as emancipated minors and were able to provide consent. The interviewers documented the receipt of verbal informed consent on the individual consent forms. The women were interviewed by trained female interviewers using paper questionnaires at private locations within or close to their residence. This study used the de-identified public-use versions of the datasets.

## Results

### Sample characteristics

The socio-demographic characteristics of the women at both surveys are shown in Table 1. The 9933 women included in this study resided in households located in 480 clusters with a range of 5–63 women per cluster. The median number of women per cluster was 24. The majority of the women were aged less than 35 years, had secondary and higher education, were currently in union, and had one or more children. During the four-year study period, 26% of the women were never in-union, 63% were ever in-union, while 11% joined a union [data not shown]. One half of the women were Muslims while the other half were mainly Christians with less than 1% at both surveys reporting indigenous or no religious affiliation. The proportions of the households grouped under the wealth quintiles at both time points were about the same as expected due to the method of calculation. The women resided in six cities with the highest proportion living in Kaduna and the lowest proportion living in Benin at baseline. There was minimal migration across the cities during the study period.

**Table 1:** Demographic characteristics of reproductive-age women living in six cities in Nigeria

	2010/2011 survey	2014 survey
	Weighted %	Weighted %
Age in years		
15–24	35.0	23.1
25–34	37.1	38.5
35–49	27.9	38.4
Education		
None/Quranic	11.7	9.9
Primary	14.8	15.1
Secondary	50.6	43.5
Higher	22.9	31.5
Union status		
Currently in-union	65.2	71.5
Not currently in-union	34.8	28.5
Religion		
Muslim	53.5	50.7
Non-Muslim (Christian, traditional, none)	46.5	49.3
Parity		
0 children	33.9	26.0
1–4 children	46.1	49.1
5 or more children	20.0	24.9
Household wealth		
Poorest	16.6	19.0
Poor	18.8	19.5
Middle	20.4	20.5
Rich	22.6	21.0
Richest	21.6	20.0
City of residence		
Abuja	12.5	13.0
Benin	10.5	13.1
Ibadan	18.0	20.2
Ilorin	16.4	15.5
Kaduna	27.1	25.5
Zaria	15.5	12.7
Number of women	9933	
Number of clusters	480	
Mean number of women per cluster (standard deviation)	26.7 (11.4)	
Median number of women per cluster (range	24 (5–63)	

Table 2 shows the distribution of the gender-equitable attitudes of the women measured at the individual and neighborhood levels in both surveys. For the gender-equitable attitudes towards wife beating, the proportion of women reporting high levels of the gender-equitable attitudes towards wife beating increased from 67 to 81% during the study period while at the neighborhood-level, the proportion of women in neighborhoods classified as having high levels of gender-equitable attitudes towards wife beating also increased from 36 to 64% during the study period. For the gender-equitable attitudes towards household decision-making score, the proportion of women reporting high levels of the gender-equitable attitudes increased from 44 to 63% within the four-year period. Similar results were noted at the neighborhood-level where the proportion of women in neighborhoods classified as having high levels of gender-equitable attitudes towards household decision-making increased from 51 to 73%. The proportion of women reporting high levels of gender-equitable attitudes towards couples’ family planning decisions increased from 40% at baseline to 58% at endline while the proportion of women who live in neighborhoods classified as having high levels of these gender-equitable attitudes increased from 29% at baseline to 66% at endline. Lastly, the proportion of women reporting high levels of the gender-equitable attitudes towards family planning self-efficacy increased from 44 to 66% by endline; likewise, the proportion of women in neighborhoods classified as having high levels of these attitudes also increased 26% to 66% within the study period. The absolute number and proportion of women with change, or lack thereof, in the gender-equitable norms during the study period is shown in Additional file 1: Table S1.

**Table 2:** Distribution of gender-equitable attitudes among women living in six cities in Nigeria

	2010/2011 survey		2014 Survey	
	Weighted %		Weighted %	
Gender-equitable attitudes towards:	Individual	Neighborhood	Individual	Neighborhood
Wife beating				
Low level (score = 0–6)	32.8	64.4	19.1	36.5
High level (score = 7)	67.2	35.6	80.9	63.5
Household decision-making				
Low level (score = 0–2)	55.9	49.5	37.4	26.9
High level (score = 3–4)	44.1	50.5	62.6	73.1
Couples’ family planning decisions				
Low level (score = 0–6)	60.4	71.1	42.2	34.3
High level (score = 7–9)	39.6	28.9	57.8	65.7
Family planning efficacy				
Low level (score = 0–5)	56.0	74.1	33.6	34.4
High level (score = 6–8)	44.0	25.9	66.4	65.6

**Fig. 3:**
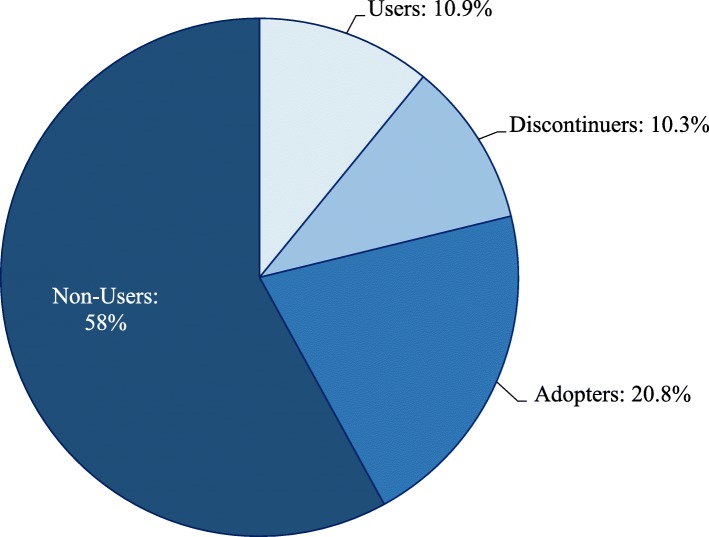
Modern contraceptive use pattern among women aged 15–49 years in Nigerian cities

Approximately 21% of the women reported using a modern contraceptive at baseline; by endline, this proportion increased to 32% – an 11 percentage-points increase within a four-year period. At baseline, the most commonly reported modern contraceptives were male condoms (8.2%), injections (4.9%), daily pills (2.4%), intrauterine device (2.1%), lactational amenorrhea (1.6%), and emergency pills (1.2%); about 0.6% of the women reported using other modern contraceptives including female or male sterilization, implants, female condoms, diaphragms, or spermicides. At endline, the commonly reported modern contraceptives were male condoms (9.6%), injections (7.6%), lactational amenorrhea (3.4%), daily pills (3.3%), intrauterine device (2.9%), implants (2.6%), and emergency pills (1.6%); about 1% reported using other modern contraceptives. Figure 3 shows the distribution of the modern contraceptive use pattern within the study period. At both surveys, 58% of the women did not report using modern contraceptives (non-users) while 11% reported using modern contraceptives (users); 21% did not use at baseline but started using by endline (adopters) while 10% used at baseline but discontinued use by endline (discontinuers).

### Relationships between gender-equitable attitudes and modern contraceptive use pattern

The associations between the gender-equitable attitudes and modern contraceptive use pattern between surveys were assessed using multilevel multinomial logistic regression models that included both the individual and neighborhood levels of the attitudes controlling for socio-demographic factors. We assessed whether the change, or lack thereof, in the gender-equitable attitudes between surveys were associated with varying modern contraceptive use patterns. However, only the following comparisons are presented: a) using a modern contraceptive compared to not using a modern contraceptive at both surveys (i.e. users versus non-users); b) adopting a modern contraceptive between surveys compared to not using a modern contraceptive at both surveys (i.e. adopters versus non-users); and c) discontinuing a modern contraceptive between surveys compared to using a modern contraceptive at both surveys (i.e. discontinuers versus users). Additional file 2: Table S2 shows the association between the women's gender-equitable attitudes during baseline survey and their modern contraceptive use by endline survey.

#### Gender-equitable attitudes towards wife beating and modern contraceptive use pattern

Table 3 shows the estimated odds ratios from the multilevel models of gender-equitable attitudes towards wife beating on modern contraceptive use pattern. No significant associations were observed between the individual-level or neighborhood-level attitudes and modern contraceptive use patterns.

**Table 3:** Gender-equitable attitudes towards wife beating and modern contraceptive use pattern

	User vs. Non-User	Adopter vs. Non-User	Discontinuer vs. User
	OR (95% CI)	OR (95% CI)	OR (95% CI)
Individual-level attitudes			
Low to Low	reference	reference	reference
Low to High	0.98 (0.70–1.37)	1.16 (0.92–1.47)	0.97 (0.64–1.48)
High to Low	1.06 (0.73–1.55)	1.10 (0.84–1.44)	1.15 (0.72–1.85)
High to High	1.12 (0.81–1.55)	1.12 (0.89–1.41)	0.97 (0.65–1.46)
Neighborhood-level attitudes			
Low to Low	reference	reference	reference
Low to High	0.97 (0.78–1.20)	0.97 (0.82–1.15)	0.89 (0.69–1.14)
High to Low	1.19 (0.84–1.69)	1.11 (0.84–1.47)	0.72 (0.46–1.11)
High to High	0.92 (0.73–1.16)	0.98 (0.82–1.18)	1.10 (0.84–1.45)

*OR* Odds Ratio, *CI* Confidence Interval; Models included individual and neighborhood exposure variables controlling for age, education, marital history, religion, parity, household wealth, and city of residence

#### Gender-equitable attitudes towards household decision-making and modern contraceptive use pattern

Table 4 shows the estimated odds ratios from the multilevel models of gender-equitable attitudes towards household decision-making on modern contraceptive use pattern. At the individual-level, having high levels of gender-equitable attitudes towards household decision-making at endline (i.e. switching from low to high levels or remaining at high levels compared to remaining at low levels) is associated with an increase in the relative probability of using a modern contraceptive at both surveys compared to not using a modern contraceptive at both surveys (OR: 1.38–1.60; *p* < 0.01). Similarly, having high levels of gender equitable attitudes towards household decision-making at endline is associated with a greater relative probability of adopting a modern contraceptive between surveys compared to not using a modern contraceptive at both surveys (OR: 1.17–1.23; *p* < 0.05). On the other hand, having high levels of gender-equitable attitudes towards household decision-making compared to having low levels at both time points is associated with a lower relative probability of discontinuing a modern contraceptive between surveys compared to using a modern contraceptive at both time points (OR: 0.73; 95% CI: 0.55–0.98). A negative change (high to low levels) at the individual-level was not significantly associated with modern contraceptive use pattern (*p* > 0.05).

**Table 4:** Gender-equitable attitudes towards household decision-making and modern contraceptive use pattern

	User vs. Non-User	Adopter vs. Non-User	Discontinuer vs. User
	OR (95% CI)	OR (95% CI)	OR (95% CI)
Individual-level attitudes			
Low to Low	reference	reference	reference
Low to High	1.38 (1.09–1.75)	1.17 (1.02–1.36)	0.81 (0.60–1.09)
High to Low	1.24 (0.93–1.64)	1.03 (0.85–1.24)	0.88 (0.62–1.24)
High to High	1.60 (1.26–2.03)	1.23 (1.05–1.45)	0.73 (0.55–0.98)
Neighborhood-level attitudes			
Low to Low	reference	reference	reference
Low to High	2.28 (1.61–3.23)	1.28 (1.03–1.61)	0.58 (0.38–0.89)
High to Low	2.23 (1.36–3.67)	2.00 (1.38–2.89)	0.72 (0.40–1.30)
High to High	2.66 (1.88–3.76)	1.58 (1.24–1.99)	0.54 (0.35–0.82)

*OR* Odds Ratio, *CI* Confidence Interval; Models included individual and neighborhood exposure variables controlling for age, education, marital history, religion, parity, household wealth, and city of residence

Controlling for the individual-level values, neighborhood-level attitudes were also associated with modern contraceptive use pattern between surveys. Compared to women who lived in neighborhoods that had low levels of gender-equitable attitudes towards household decision-making at both time points, those who lived in neighborhoods that had high levels of gender-equitable attitudes at endline had higher relative probabilities of using a modern contraceptive at both surveys (OR: 2.28–2.66; *p* < 0.01) or adopting a modern contraceptive between surveys (OR: 1.28–1.58; *p* < 0.05) as opposed to not using a modern contraceptive at both surveys. Women who lived in neighborhoods that started out at high levels but switched to low levels by endline also had higher relative probabilities of being modern contraceptive users and adopters compared to being non-users between surveys (OR: 2.00–2.23; *p* < 0.001). However, living in neighborhoods that had positive changes in the levels of gender-equitable attitudes towards household decision-making compared to living in neighborhoods that remained at low levels at both time points was associated with lower relative probabilities of discontinuing modern contraceptives between the surveys compared to using modern contraceptives at both surveys (OR: 0.54–0.58; p < 0.05).

#### Gender-equitable attitudes towards couples’ family planning decisions and modern contraceptive use pattern

Table 5 shows the estimated odds ratios from the multilevel models of gender-equitable attitudes towards couples’ family planning decisions on modern contraceptive use pattern. Both the individual level and neighborhood-level gender-equitable attitudes were associated with the modern contraceptive use pattern. Specifically, compared to women whose gender-equitable attitudes towards couples’ family planning decisions remained low during the study period, those whose attitudes changed from low to high levels had higher relative probability of adopting a modern contraceptive between surveys as opposed to not using a modern contraceptive at both surveys (OR: 1.37; 95% CI: 1.19–1.57). Women who remained at high levels compared to those that remained at low levels during the study period had higher relative probability of adopting a modern contraceptive between surveys or using a modern contraceptive at both surveys as opposed to not using modern contraceptive at both surveys (OR: 1.54–2.34; *p* < 0.001); these women also had lower relative probability of discontinuing a method during the study period (OR: 0.73; 95% CI: 0.55–0.97). Switching from high to low levels compared to remaining at low levels at both time points was associated with higher relative probability of using a modern contraceptive compared to not using a modern contraceptive at both surveys (OR: 1.49; 95% CI: 1.16–1.91). No such associations were observed for adopter versus non-user status or discontinuer versus user status for the women that had negative changes (*p* > 0.05).

**Table 5:** Gender-equitable attitudes towards couples’ family planning decisions and modern contraceptive use pattern

	User vs. Non-User	Adopter vs. Non-User	Discontinuer vs. User
	OR (95% CI)	OR (95% CI)	OR (95% CI)
Individual-level attitudes			
Low to Low	reference	reference	reference
Low to High	1.13 (0.91–1.41)	1.37 (1.19–1.57)	1.04 (0.78–1.38)
High to Low	1.49 (1.16–1.91)	1.01 (0.83–1.22)	1.18 (0.87–1.61)
High to High	2.34 (1.87–2.93)	1.54 (1.31–1.82)	0.73 (0.55–0.97)
Neighborhood-level attitudes			
Low to Low	reference	reference	reference
Low to High	1.15 (0.92–1.45)	1.10 (0.93–1.31)	0.72 (0.55–0.94)
High to Low	1.38 (0.92–2.05)	1.16 (0.83–1.62)	0.90 (0.57–1.42)
High to High	1.47 (1.13–1.90)	1.12 (0.90–1.39)	0.62 (0.45–0.84)

*OR* Odds Ratio, *CI* Confidence Interval; Models included individual and neighborhood exposure variables controlling for age, education, marital history, religion, parity, household wealth, and city of residence

Additionally, controlling for the individual-level attitudes, women who lived in neighborhoods that remained at high levels compared to low levels at both time points had higher relative probability of using a modern contraceptive compared to not using a modern contraceptive at both surveys (OR: 1.47; 95% CI: 1.13–1.90) and lower relative probability of discontinuing a modern contraceptive between surveys versus using a modern contraceptive at both surveys (OR: 0.62; 0.45–0.84). Likewise, women who lived in neighborhoods that switched from low to high levels compared to those who remained at low levels had lower relative probability of discontinuing a modern contraceptive between surveys versus using a modern method at both surveys (OR: 0.72; 95% CI: 0.55–0.95). Living in neighborhoods that switched from high to low levels as compared to neighborhoods that remained at low levels did not significantly affect the relative probability of being a modern contraceptive user, adopter, or discontinuer during the study period (*p* > 0.05).

#### Gender-equitable attitudes towards family planning efficacy and modern contraceptive use pattern

As shown in Table 6, the individual-level gender-equitable attitudes towards family planning self-efficacy were associated with modern contraceptive use pattern during the study period. Women whose levels of gender-equitable attitudes changed from low to high levels compared to those who remained at low levels at both time points had higher relative probability of using modern contraceptives at both surveys (OR: 3.33; 95% CI: 2.42–4.60) or adopting modern contraceptives between surveys (OR: 2.37; 95% CI: 2.03–2.78) compared to not using modern contraceptives at both surveys. These women also had lower relative probability of discontinuing a modern contraceptive between surveys compared to those using a modern contraceptive at both surveys (OR: 0.42; 95% CI: 0.28–0.63). Similar associations were observed among women whose attitudes remained at high levels as opposed to low levels at both time points. Interestingly, women who started off at high levels of the attitudes but changed to low levels by endline also had higher relative probabilities of using modern contraceptives at both surveys (OR: 3.14; 95% CI: 2.18–4.53) or adopting modern contraceptives between surveys (OR: 1.41; 95% CI: 1.14–1.74) compared to not using modern contraceptives at both surveys. No significant difference was observed among these women for discontinuing a modern contraceptive compared to using a modern contraceptive at both time points (OR: 1.07; 95% CI: 0.70–1.64).

**Table 6:** Gender-equitable attitudes towards family planning efficacy and modern contraceptive use pattern

	User vs. Non-User	Adopter vs. Non-User	Discontinuer vs. User
	OR (95% CI)	OR (95% CI)	OR (95% CI)
Individual-level attitudes			
Low to Low	reference	reference	reference
Low to High	3.33 (2.42–4.60)	2.37 (2.03–2.78)	0.42 (0.28–0.63)
High to Low	3.14 (2.18–4.53)	1.41 (1.14–1.74)	1.07 (0.70–1.65)
High to High	8.24 (6.02–11.27)	2.91 (2.44–3.45)	0.43 (0.30–0.63)
Neighborhood-level attitudes			
Low to Low	reference	reference	reference
Low to High	1.12 (0.88–1.43)	1.10 (0.91–1.32)	1.11 (0.83–1.49)
High to Low	0.54 (0.32–0.93)	1.01 (0.67–1.15)	1.60 (0.86–2.94)
High to High	1.38 (1.06–1.79)	1.13 (0.91–1.40)	0.91 (0.66–1.25)

*OR* Odds Ratio, *CI* Confidence Interval; Models included individual and neighborhood exposure variables controlling for age, education, marital history, religion, parity, household wealth, and city of residence

The neighborhood-level attitudes towards family planning self-efficacy were only associated with user versus non-user status but not with adopter versus non-user status or discontinuer versus user status. Specifically, irrespective of their own attitudes, women who lived in neighborhoods that remained at high levels had higher relative probability of using a modern method compared to not using a modern method at both surveys (OR: 1.38; 95% CI: 1.06–1.79) as opposed to women who lived in neighborhoods that remained at low levels at both surveys. Additionally, compared to women who lived in neighborhoods that remained at low levels, women who lived in neighborhoods that switched from high to low levels had lower relative probability of using modern methods compared to not using modern contraceptives at both time points (OR: 0.54; 95% CI: 0.32–0.93). Other observed associations were not statistically significant (*p* > 0.05).

The estimated between-cluster variations from all the models were used to calculate the intra-class correlation (ICC) and the proportion of the variation explained by each of the full models. With an estimated neighborhood-level variance of 0.45 from the null model, the calculated ICC was 0.12 meaning that 12% of the variance in the pattern of modern method use in this study was explained by the variation between neighborhoods. The residual between-cluster variance for the models with each of the four gender-equitable attitudes ranged from 0.17 to 0.20 – decreasing from 0.45 in the null model. Likewise, the ICC from the full models decreased from 0.12 to 0.05–0.06. These reductions implied that the variables included in the models explained about 55–61% of the variation between neighborhoods as it pertains to the modern contraceptive use pattern in this study [data not shown].

## Discussion

The global mandate to ensure gender equality and to improve women’s health is evident in their inclusion as Sustainable Development Goals to be achieved by 2030. The United Nations states that both gender equality and family planning are basic human rights and have the potential to improve women’s health [26]. Empirical evidence supporting this mandate is sparse especially in sub-Saharan Africa where maternal health indices remain poor. In addition, the rapid urbanization occurring in Nigeria and all of sub-Saharan Africa highlights the importance of understanding the role gender norms play in women’s contraceptive behavior in urban contexts. This study provides information on the modern contraceptive use among women living in urban Nigeria and the association of their gender norms with their use of modern contraceptives over time. During the four-year study period, the prevalence of modern contraceptive use increased by 11 percentage-points. The proportion of women reporting high levels of the gender-equitable attitudes increased within the study period at both the individual and neighborhood levels. Significant associations were found between the gender-equitable attitudes and modern contraceptive adoption, continuation, and discontinuation. This evidence supports the hypothesis that gender equality contributes to women’s health.

Looking closely at the dimensions of gender norms assessed, this study found no significant associations between the individual and neighborhood levels of gender-equitable attitudes towards wife beating and modern contraceptive use pattern. This finding was surprising as previous studies found significant associations between tolerant attitudes towards wife beating and modern contraceptive use and even with other maternal health services in sub-Saharan Africa including Nigeria [16, 31, 45, 46]. This discrepancy could be explained by the fact that this study used longitudinal data from urban women unlike previous studies that have mainly used cross-sectional data and/or included both urban and rural women in their analyses. Additionally, other studies measured attitudes towards wife beating using four scenarios, which were a subset of the seven scenarios used in this study. Although the proportion of women who reported intolerant attitudes towards wife beating at baseline was similar to what was found in previous studies that used only cross-sectional data (about 60%) [31, 47, 48]; this study found that by endline the proportion reporting intolerant attitudes towards wife beating became almost universal at 81%. This universality may also be a reason no significant associations with modern contraceptive use pattern were found. Also, the attitudes towards wife beating may have direct effects on the experience of intimate partner violence more so than on modern contraceptive use. Previous studies found mixed results on the association between the experience of gender-based violence (intimate partner violence) and modern contraceptive use. Some studies found positive associations [49, 50] while others found negative associations [51]. It could be that the different forms of intimate partner violence (sexual, physical, or emotional violence) have varying effects on modern contraceptive use. Thus, further research is needed to delineate the causal pathways between intimate partner violence (attitudes and/or experience) and modern contraceptive use.

This study also found that gender-equitable attitudes towards household decision-making were positively associated with adoption and continued use of modern contraceptives at both the individual and neighborhood levels. This finding supports the current evidence on the effects of having household decision-making power on women’s health [16, 18, 31, 45, 46, 49]. In addition, this study provides evidence that having a positive increase in and/or remaining at high levels of gender-equitable attitudes towards women having household decision-making power is associated with modern contraceptive adoption and continued use over time. Further, irrespective of personal beliefs, living in neighborhoods with high levels of these gender-equitable attitudes is positively associated with modern contraceptive adoption and continued use, and negatively associated with modern contraceptive discontinuation over time. In addition to the favorable influence of having household decision-making power on modern contraceptive use, this study found that women who believe that men and women should have control over their decision to practice family planning had higher probability of modern contraceptive adoption and continued use and lower probability of modern contraceptive discontinuation. Similar results were also observed among women who live in neighborhoods that held such beliefs. Lastly, this study supports previous findings that women’s family planning self-efficacy is positively associated with use of modern contraceptives [52–54].

This study is not without limitations. The first limitation is based on how urban community was defined. Although previous studies used census enumeration areas (clusters) as communities in the study of contextual effects on reproductive health outcomes [55–58], the assumption that these clusters are appropriate proxies for urban communities may be unfounded. The census clusters often consist of local communities in rural areas and usually a block of households in urban areas. These clusters are usually not of equal sizes or boundaries. The use of these clusters may be appropriate in rural areas where people living in the same local community are related or have many characteristics in common. This may not be the case in urban areas where individuals come from diverse backgrounds and cultures. Urban residents may interact with people in the same office or school more than their neighbors. The lack of associations between some of the neighborhood-level attitudes and modern contraceptive use pattern could be a result of this lack of connectivity. Future research should focus on understanding what a ‘community’ means in urban contexts.

Another limitation of this study was the proportion of the sample lost to follow-up; about one-third of the baseline sample was not re-interviewed at endline. A sensitivity analysis showed that unmarried, younger, and poor women were more likely to be lost to follow-up. Thus, it is possible that the associations found in this study may be different for these women. More analyses may be required to understand the effects of contextual factors on sub-populations based on age, marital status, educational attainment, or wealth status to help inform the role of this loss to follow-up. Additionally, this study used data from two time points in a four-year period in assessing the modern contraceptive use pattern. The contraceptive use pattern in this time period could be more complex than was observed; for example, the non-users may have used at some point in the study period but not at the time of the endline survey. The use of the family planning calendar data is likely to be more appropriate in assessing such patterns. Although the use of multilevel models in this study was warranted because of the nested observations (in communities and over time), certain assumptions of the models may have been violated. One such assumption is that the group-level effects are not correlated with any of the included variables. This phenomenon poses endogeneity issues; thus, causality could not be assessed.

In spite of these limitations, the results of this study add to existing literature on the effects of gender norms on women’s health. Inequitable gender norms act as social constraints that decrease women’s ability to engage in healthy reproductive behaviors and/or seek services for their reproductive health needs. Thus, family planning programs that incorporate actions or components geared towards ensuring gender equality, or promoting gender-equitable norms, are likely to see increases in the proportion of women who adopt modern contraceptives and decreases in the proportion that discontinue modern contraceptives. According to Keheler & Franklin (2008), such interventions can be implemented at the level of the individual (downstream intervention), community (midstream intervention), or population (upstream intervention) [17]. A combination of interventions at multiple levels is likely to produce synergistic program effects and may work better in communities where gender inequality exists. The downstream intervention may involve reaching women in the homes, at their workplace, or within their communities through the use of community health workers or peer educators. The midstream intervention may entail community advocacy and/or media campaigns. Evaluation of previous media campaigns on family planning use in some Nigerian cities showed positive effects on attitudes towards use and adoption of modern contraceptives [59, 60]. Thus, including female empowerment messaging in such campaigns has the potential to increase gender-equitable norms, which will in turn, improve modern contraceptive use. Strategies for national (upstream) interventions may include policies to increase female education and empowerment, and/or reduce gender-based practices that harm women (e.g., violence against women, female genital mutilation). Thus, there is a need to develop, implement, and evaluate multi-strategy programs that combine all three levels of interventions aimed at eliminating gender inequality in Nigeria.

## Conclusion

This study provides evidence that gender norms are associated with modern contraceptive adoption, continuation, and discontinuation among reproductive-aged women living in urban Nigeria. Thus, programs aimed at reducing harmful gender norms and promoting gender-equitable norms have the potential to increase the prevalence of modern contraceptive use, thereby reducing the maternal mortality rate due to unintended pregnancy.

## Additional files

Additional file 1: **Table S1.** Proportion of women with change, or lack thereof, in the gender-equitable norms during the study period. The absolute number and proportion of women with change, or lack thereof, in the gender-equitable norms during the study period. (DOCX 16 kb)

Additional file 2: **Table S2.** Association between baseline gender-equitable attitudes and modern contraceptive use at endline. Bivariate analyses of the women’s gender-equitable attitudes at baseline survey and their modern contraceptive use at endline survey. (DOCX 16 kb)

## Abbreviations

CI: Confidence Interval
CPR: Contraceptive Prevalence Rate
GLLAMM: Generalized Linear Latent and Mixed Models
ICC: Intra-class Correlation Coefficient
MDG: Millennium Development Goals
MLE Project: Measurement, Learning and Evaluation Project
MMR: Maternal Mortality Ratio
NDHS: Nigeria Demographic and Health Surveys
NURHI: Nigerian Urban Reproductive Health Initiative
OR: Odds Ratio
SDG: Sustainable Development Goals
URHI: Urban Reproductive Health Initiative

## Footnotes

1 The modern contraceptive methods include female and male sterilization, intrauterine device, implants, injections, daily pills, male and female condoms, diaphragm, foam/jelly, lactational amenorrhea method, and emergency contraception.
