# Supplementary material for: Gender norms and modern contraceptive use in urban Nigeria: a multilevel longitudinal study
Source: BMC Womens Health. 2018 Oct 29;18:178. doi: 10.1186/s12905-018-0664-3 (PMC6206649; doi:10.1186/s12905-018-0664-3)
Supplement: Supplementary file 1 — Table S1. Proportion of women with change, or lack thereof, in the gender-equitable norms during the study period. The absolute number and proportion of women with change, or lack thereof, in the gender-equitable norms during the study period. (DOCX 16 kb) [file 12905_2018_664_MOESM1_ESM.docx]

Additional file 1: Table S1 Proportion of women with change, or lack thereof, in the gender-equitable norms during the study period

|  | Wife-beating attitudes  N (%) | Household decision-making attitudes  N (%) | Couples’ family planning decisions attitudes  N (%) | Family planning efficacy attitudes  N (%) |
| --- | --- | --- | --- | --- |
| Individual-level attitudes | | | | |
| Low to Low | 640 (6.5) | 2882 (29.0) | 2906 (29.3) | 2648 (26.7) |
| Low to High | 2714 (27.3) | 3022 (30.4) | 3314 (33.3) | 3150 (31.7) |
| High to Low | 1206 (12.1) | 1326 (13.4) | 1397 (14.1) | 1185 (11.9) |
| High to High | 5373 (54.1) | 2703 (27.2) | 2316 (23.3) | 2950 (29.7) |
| Neighborhood-level attitudes | | | | |
| Low to Low | 2640 (26.6) | 3048 (30.7) | 3475 (35.0) | 3776 (38.0) |
| Low to High | 4042 (40.7) | 2474 (24.9) | 3810 (38.3) | 3725 (37.5) |
| High to Low | 641 (6.5) | 389 (3.9) | 416 (4.2) | 281 (2.8) |
| High to High | 2610 (26.2) | 4022 (40.5) | 2232 (22.5) | 2151 (21.7) |
