# Supplementary material for: Gender norms and modern contraceptive use in urban Nigeria: a multilevel longitudinal study
Source: BMC Womens Health. 2018 Oct 29;18:178. doi: 10.1186/s12905-018-0664-3 (PMC6206649; doi:10.1186/s12905-018-0664-3)
Supplement: Supplementary file 2 — Table S2. Association between baseline gender-equitable attitudes and modern contraceptive use at endline. Bivariate analyses of the women’s gender-equitable attitudes at baseline survey and their modern contraceptive use at endline survey. (DOCX 16 kb) [file 12905_2018_664_MOESM2_ESM.docx]

Additional file 2: Table S2 Association between baseline gender-equitable attitudes and modern contraceptive use at endline.

| Reference Group: Non-Users | Adopters: Unadjusted OR (95% CI) | Discontinuers: Unadjusted OR (95% CI) | Users: Unadjusted OR (95% CI) |
| --- | --- | --- | --- |
| Individual-level attitudes to wife beating | 1.01 (0.99-1.04) | 1.06 (1.02-1.10)** | 1.07 (1.03-1.1)*** |
| Neighborhood-level attitudes to wife beating attitudes | 1.02 (0.96-1.07) | 0.94 (0.88-1.02) | 1.03 (0.95-1.11) |
|  | | | |
| Individual-level attitudes to household decisions | 1.12 (1.08-1.16)*** | 1.33 (1.27-1.40)*** | 1.43 (1.35-1.50)*** |
| Neighborhood-level attitudes to household decisions | 1.46 (1.36-1.57)*** | 2.23 (2.02-2.47)*** | 2.63 (2.37-2.92)*** |
|  | | | |
| Individual-level attitudes to couples’ fertility decisions | 1.06 (1.04-1.10)*** | 1.36 (1.31-1.42)*** | 1.39 (1.34-1.45)*** |
| Neighborhood-level attitudes to couples’ fertility decisions | 1.20 (1.14-1.26)*** | 1.70 (1.59-1.82)*** | 1.86 (1.74-2.00)*** |
|  | | | |
| Individual-level attitudes to family planning self-efficacy | 1.11 (1.08-1.13)*** | 1.51 (1.46-1.57)*** | 1.54 (1.48-1.60)*** |
| Neighborhood-level attitudes to family planning self-efficacy | 1.16 (1.11-1.20)*** | 1.61 (1.53-1.70)*** | 1.66 (1.57-1.76)*** |
